# Differences in the Association Between Alcoholic Beverage Type and Serum Urate Levels Using Standardized Ethanol Content

**DOI:** 10.1001/jamanetworkopen.2023.3398

**Published:** 2023-03-17

**Authors:** Sho Fukui, Masato Okada, Mahbubur Rahman, Hiroki Matsui, Atsushi Shiraishi, Takehiro Nakai, Hiromichi Tamaki, Mitsumasa Kishimoto, Hiroshi Hasegawa, Takeaki Matsuda, Kazuki Yoshida

**Affiliations:** 1 Immuno-Rheumatology Center, St Luke’s International Hospital, Tokyo, Japan; 2 Graduate School of Public Health, St Luke’s International University, Tokyo, Japan; 3 Department of Emergency and General Medicine, Kyorin University School of Medicine, Tokyo, Japan; 4 Clinical Research Support Office, Kameda Medical Center, Kamogawa, Japan; 5 Emergency and Trauma Center, Kameda Medical Center, Kamogawa, Japan; 6 Department of Nephrology and Rheumatology, Kyorin University School of Medicine, Tokyo, Japan

## Abstract

**Importance:**

Differences have been observed in the association of serum urate levels with consumption of different types of alcoholic beverages. However, previous studies have not standardized the unit of intake for ethanol content, and only limited types of alcoholic beverages have been evaluated.

**Objective:**

To examine differences in the association of serum urate levels with various types of alcoholic beverages when their intakes are standardized for ethanol content.

**Design, Setting, and Participants:**

This retrospective cross-sectional study was conducted using data from participants aged 20 years or older who completed a medical checkup at St Luke’s International University in Japan between October 1, 2012, and October 31, 2021. Participant demographics, blood test results, and lifestyle questionnaire data were used as covariates. Analysis was performed in December 2021.

**Exposures:**

Consumption of alcoholic beverages, including beer, sake (rice wine), shochu (Japanese spirit), wine, and whiskey.

**Main Outcomes and Measures:**

Serum urate levels were measured during the medical checkup. The beverage unit was standardized to 1 standard drink, which contained 20 g of ethanol. Multivariable linear regression including interaction terms of alcohol consumption and dominant alcoholic beverage was performed.

**Results:**

This study included 78 153 participants. Their mean (SD) age was 47.6 (12.8) years; 36 463 (46.7%) were men and 41 690 were women (53.3%). A total of 45 755 participants (58.5%) were regular alcohol drinkers. Consistent associations of serum urate levels with alcohol consumption were observed in the beer-dominant group, with β coefficients (for 1 standard drink per day) of 0.14 mg/dL (95% CI, 0.11-0.17 mg/dL; *P* < .001) for men and 0.23 mg/dL (95% CI, 0.20-0.26 mg/dL; *P* < .001) for women. A moderate increase in serum urate levels was observed in the wine-dominant group compared with a modest and nonsignificant increase in the sake-dominant group, with β coefficients (for 1 standard drink per day) for the latter group of 0.05 mg/dL (95% CI, −0.01 to 0.10; *P* = .10) for men and 0.04 mg/dL (95% CI, −0.05 to 0.14 mg/dL; *P* = .38) for women. Restricted cubic splines showed different patterns in associations of serum urate levels with ethanol intake by dominant alcoholic beverages.

**Conclusions and Relevance:**

The results of this study suggest that the extent of the association of serum urate levels with alcohol intake was different for alcoholic beverages even after ethanol content was standardized. Higher beer consumption among men and women was consistently associated with higher serum urate levels, whereas sake was not associated with changes in serum urate levels. Therefore, alcoholic beverage type, in addition to ethanol content, should be considered as a factor contributing to hyperuricemia.

## Key Points

**Question:**

Are there differences in the association of serum urate levels with consumption of alcohol, including Japanese traditional beverages, when intake is standardized for ethanol content?

**Findings:**

In this cross-sectional study using medical checkup data from 78 153 Japanese participants, differences were observed in the association of serum urate levels with alcohol consumption even after ethanol content was standardized. Consumption of beer and wine was associated with high and moderate increases in serum urate levels, respectively; in contrast, sake was associated with a modest increase in serum urate levels.

**Meaning:**

The results of this study suggest that alcoholic beverage type, in addition to ethanol content, should be considered as a contributing factor to high serum urate levels.

## Teaser

This cross-sectional study examines differences in the association of serum urate levels with consumption of alcohol, including Japanese traditional beverages such as sake and shochu, using intake standardized for ethanol content.

## Introduction

Uric acid is an end product of purine metabolism.^1^ Elevated serum urate levels are a well-known precursor of gout, which is caused by deposition of monosodium urate crystals. The prevalence of hyperuricemia has been increasing worldwide^2,3^ and has attracted increasing attention as an independent risk factor for cardiovascular disease^4^ and an associated increase in mortality.^5^ In addition, previous studies suggest that high serum urate levels are associated with hypertension,^6^ peripheral arterial disease,^7^ diabetes,^8^ and chronic kidney disease.^9,10^

Serum urate levels increase in association with intake of a broad range of foods and drinks^11,12^ and with medical conditions such as diabetes and kidney dysfunction.^13^ Alcohol consumption is the primary dietary risk factor contributing to high serum urate levels.^11^ The ethanol in alcoholic beverages plays an important role in changing serum urate levels by increasing uric acid production^14,15^ and decreasing the elimination of uric acid to urine by modulating kidney tubule function.^16^ Other ingredients in alcoholic beverages (eg, purines) can also affect serum urate levels.^17^ Therefore, the influence of serum urate levels differs by alcoholic beverage type (eg, beer, liquor, and wine).^18^

In previous studies, the ethanol content in 1 alcoholic beverage unit has not been standardized, and only limited types of alcoholic beverages have been evaluated. These limitations make it difficult to compare the influence of alcoholic beverage type regardless of ethanol content. Furthermore, the popularity of Japanese alcoholic beverages such as sake (Japanese rice wine) and shochu (Japanese spirit) is increasing worldwide. However, there are limited studies regarding the association of alcoholic beverage intake with serum urate levels.^19,20^

For this study, we used medical checkup data to examine differences in serum urate levels associated with alcoholic beverage types in Japan. We compared the association of serum urate levels with consumption of alcoholic beverages, including sake and shochu, by standardizing intake for ethanol content.

## Methods

### Study Design and Setting

This cross-sectional study was approved by the St Luke’s International University Institutional Review Board. The study was conducted based on the Declaration of Helsinki and relevant ethical guidelines for medical research in Japan. Written informed consent was waived because of the retrospective design, with options for opting out. The study followed the Strengthening the Reporting of Observational Studies in Epidemiology (STROBE) reporting guideline.

We used data from the St Luke’s Health Check-up Database (SLHCD) for October 1, 2012, to October 31, 2021. The SLHCD collects data on participant demographics and results from medical checkups performed at the Center for Preventive Medicine at St Luke’s International Hospital Tertiary Care Center in Tokyo, Japan. In Japan, all employers are required to encourage employees to undergo medical checkups, including laboratory testing, at least annually.^21^ In our medical checkups, participants were routinely asked to answer a questionnaire regarding their medical history and lifestyle factors. They also underwent a physical examination, determination of height and weight, blood testing, other assessments (electrocardiogram and chest radiography), and subsequent medical interviews to obtain recommendations for lifestyle modification.

### Study Participants and Data Collection

We included consecutive participants aged 20 years or older who had undergone a medical checkup and completed the lifestyle questionnaire. We excluded individuals who refused to participate in the research, were receiving pharmacotherapy for hyperuricemia at assessment, answered that they drink “other types of alcoholic beverages,” or reported drinking more than 10 standard alcoholic drinks per day. Individuals who consumed more than 10 alcoholic drinks per day were considered very heavy drinkers, with an average amount of daily alcohol consumption equivalent to high-intensity drinking.^22^ Heavy drinking may potentially lower serum urate levels as a result of kidney tubular dysfunction.^23,24^

We used data from the first medical checkup during the study period for each participant. Data on participant demographics (eg, age, sex, body mass index [BMI], serum urate blood test results, and estimated glomerular filtration rate [eGFR]), prespecified medical conditions, and treatment status were retrieved from the SLHCD. We used data on medication use for angina, myocardial infarction, transient ischemic attack, cerebral infarction, hypertension, diabetes, dyslipidemia, chronic kidney disease, tuberculosis, and nontuberculosis mycobacteria infections because these conditions can be treated with medications that can potentially change serum urate levels.^25^ Examples of these medications include aspirin, diuretics, calcium blockers, angiotensin receptor blockers, sodium-glucose cotransporter 2 inhibitors, statins, pyrazinamide, and ethambutol. The lifestyle questionnaires included smoking status (never, previous, or current), daily physical activity (very low, low, moderate, or high), exercise level (<1, 1-2, 3-5, or >5 days per week with at least 20 minutes of light, sweaty exercise), and frequency of dietary intake of carbohydrate (eg, rice, bread, and noodles), meat and eggs, seafood, vegetables, fruits, milk and milk products, soy, fat (eg, fried foods, animal fat, and other fatty meals), and sweets.

### Measurement of Alcohol Consumption and Definition of Dominant Alcoholic Beverage Groups

The lifestyle questionnaire asked participants whether they were regular alcohol drinkers (≥1 alcoholic beverage per week). If they answered yes, they were then asked to report the frequency of alcohol consumption (days per week) and the average amount of each alcoholic beverage type consumed. The types and amounts of alcoholic beverages were recorded based on daily consumption as the number of the following: small cans (350 mL), large cans (500 mL), or large bottles (633 mL) of beer; gou (Japanese traditional alcohol units) of sake; glasses of shochu (Japanese spirit; usually 100 mL/glass); glasses of wine (usually 120 mL); a single shot of whiskey (30 mL); and glasses of other beverages.

We calculated the ethanol content in these beverages using the standardized alcohol content as follows: 5% for beer, 15% for sake, 25% for shochu, 12% for wine, and 40% for whiskey. The ethanol content for a unit of each alcoholic beverage was converted to a standard drink, which included the same ethanol content to make the comparison easier. Because there is no universal standard drink unit in Japan, we defined a standard drink as a unit that includes 20 g of pure ethanol based on the recommended upper limit of daily alcohol consumption by the Japanese Ministry of Health, Labor, and Welfare. One standard drink is equivalent to 500 mL of beer, 167 mL (0.93 gou) of sake, 100 mL of shochu (approximately 1 glass), 208 mL of wine (approximately 1.7 glasses), or 62.5 mL of whiskey (approximately 2 shots).

A dominant alcoholic beverage was defined as an alcoholic beverage type that accounted for 75% or more of total ethanol consumption. Participants were classified according to their dominant alcoholic beverage type. If participants had no dominant beverage type, they were classified as a mixed-alcohol group.

### Statistical Analysis

First, we described the characteristics of participants overall and by groups of dominant alcoholic beverages. Then we performed multivariable linear regression analyses to evaluate the association of serum urate levels (in milligrams per decaliter) with alcoholic beverage consumption. In these analyses, serum urate level was set as a dependent variable, which was normally distributed; independent variables of interest included total alcohol consumption and consumption of each alcoholic beverage (standard drinks per day). In addition, we evaluated the association of alcohol consumption with hyperuricemia (defined as serum urate levels ≥7 mg/dL for men and ≥6 mg/dL for women, based on previous studies^26^) using a multivariable logistic regression model. In these analyses, age, sex, BMI, eGFR, medication use, smoking status, daily physical activity, exercise level, and dietary questionnaire results were used as covariates for adjustments. Each lifestyle or dietary factor (described in eTables 1 and 2 in Supplement 1) was adjusted as a categorical variable, with the answer indicating the most infrequent factor as a reference. In addition, the amounts of other alcoholic beverages were also adjusted when we focused on a specific type of alcoholic beverage as an exposure of interest. All analyses were stratified by sex because a large difference in serum urate levels between men and women has been observed.^3^

Second, we directly compared the association of serum urate levels with alcohol intake in individual dominant alcoholic beverage groups. Considering that ethanol content is the most important factor for increased serum urate levels, we included interaction terms of total alcohol consumption and dominant alcoholic beverage in the multivariable linear model using the same covariates as in previous analyses. Serum urate levels were then estimated using daily alcohol consumption at the mean values of other covariates. The association of estimated serum urate level with daily alcohol consumption was visualized.

Thereafter, we developed more flexible models to assess the association of serum urate levels with alcohol consumption among each dominant group using a restricted cubic spline to evaluate the potential departure from a linear association. We used the mkspline function in Stata 17, version 17.1 (StataCorp LLC), with percentile knots (5th, 35th, 65th, and 95th percentiles). In addition, analyses with fixed knots of 1, 2, 3, and 4 standard drinks per day were performed as sensitivity analyses.

*P* < .05 in 2-tailed tests was considered significant for all analyses. Analysis was performed in December 2021.

## Results

### Participant Characteristics

A total of 78 153 participants were included in this study (eFigure 1 in Supplement 1). Their mean (SD) age was 47.6 (12.8) years; 36 463 (46.7%) were men and 41 690 (53.3%) were women. A total of 45 755 participants (58.5%) were regular alcohol drinkers, and their mean (SD) consumption of total alcoholic beverages was 1.10 (1.13) standard drinks per day. Among men, there were 21 377 beer drinkers (58.6%), 3090 sake drinkers (8.5%), 5900 shochu drinkers (16.2%), 4883 wine drinkers (13.4%), and 2142 whiskey drinkers (5.9%). Among women, there were 12 137 beer drinkers (29.1%), 945 sake drinkers (2.3%), 1515 shochu drinkers (3.6%), 7722 wine drinkers (18.5%), and 523 whiskey drinkers (1.3%). These drinkers were not mutually exclusive, as 1 participant could drink several types of alcoholic beverages.

In contrast, the dominant alcoholic beverage groups were mutually exclusive. Beer-dominant drinking was most common among both men and women. Men more frequently consumed sake, shochu, and whiskey compared with women (Table 1), whereas wine was more common among women (Table 2). Results of the lifestyle and dietary questionnaires are summarized in eTables 1 and 2 in Supplement 1, respectively.

**Table 1.** Characteristics of Male Participants

Characteristic	Total (n = 36 463)	Not a regular drinker (n = 9527)	Dominant alcoholic beverage					Mixed alcohol (n = 7810)
			Beer (n = 12 711)	Sake (n = 1384)	Shochu (n = 2343)	Wine (n = 2088)	Whiskey (n = 600)	
Age, mean (SD), y	47.9 (12.8)	47.4 (14.6)	45.1 (11.8)	56.3 (12.6)	52.4 (11.4)	50.7 (11.5)	48.4 (12.4)	49.4 (11.2)
BMI, median (IQR)	23.3 (21.6-25.3)	23.1 (21.3-25.4)	23.1 (21.4-25.0)	23.5 (21.7-25.4)	23.9 (22.2-26.0)	23.3 (21.7-25.3)	23.8 (22.0-25.9)	23.6 (22.0-25.6)
Frequency of alcohol intake, median (IQR), times/wk	3.0 (0-6.0)	0	3.0 (2.0-6.0)	5.0 (3.0-7.0)	6.0 (3.0-7.0)	4.0 (2.0-6.0)	5.0 (3.0-7.0)	5.0 (3.0-7.0)
Amount of alcohol, No. of participants, drinks/d								
Not a regular drinker	9527 (26.1)	9527 (100)	0	0	0	0	0	0
0 to <1	14 132 (38.8)	0	9685 (76.2)	514 (37.1)	558 (23.8)	1311 (62.8)	337 (56.2)	1727 (22.1)
1 to <2	6827 (18.7)	0	2175 (17.1)	387 (28.0)	513 (21.9)	508 (24.3)	162 (27.0)	3082 (39.5)
2 to <3	3575 (9.8)	0	657 (5.2)	362 (26.2)	511 (21.8)	175 (8.4)	67 (11.2)	1803 (23.1)
≥3	2402 (6.6)	0	194 (1.5)	121 (8.7)	761 (32.5)	94 (4.5)	34 (5.7)	1198 (15.3)
Total alcohol, mean (SD), drinks/d	0.99 (1.23)	0	0.78 (0.77)	1.61 (1.06)	2.53 (1.66)	1.02 (0.92)	1.16 (1.04)	1.95 (1.32)
Beer	0.44 (0.63)	0	0.77 (0.75)	0.14 (0.25)	0.19 (0.33)	0.03 (0.14)	0.03 (0.14)	0.72 (0.53)
Sake	0.10 (0.42)	0	0 (0.02)	1.47 (0.93)	0 (0.03)	0 (0.02)	0	0.22 (0.52)
Shochu	0.28 (0.81)	0	0 (0.03)	0 (0.04)	2.32 (1.49)	0 (0.02)	0	0.59 (0.88)
Wine	0.12 (0.40)	0	0 (0.03)	0 (0.03)	0.01 (0.07)	0.99 (0.85)	0 (0.02)	0.29 (0.52)
Whiskey	0.05 (0.26)	0	0 (0.03)	0 (0.03)	0.01 (0.06)	0 (0.05)	1.13 (0.99)	0.13 (0.36)
Serum urate level, mean (SD), mg/dL	6.33 (1.20)	6.18 (1.20)	6.33 (1.18)	6.32 (1.18)	6.56 (1.24)	6.33 (1.21)	6.54 (1.33)	6.44 (1.21)
Kidney function (eGFR), mean (SD), mL/min/1.73 m^2^	108.9 (27.9)	107.2 (30.8)	111.4 (26.2)	98.9 (27.1)	107.6 (28.5)	105.7 (26.5)	111.0 (28.7)	109.6 (26.2)
Medication use indication, No. of participants (%)								
Hypertension	4166 (11.4)	969 (10.2)	1001 (7.9)	273 (19.7)	514 (21.9)	243 (11.6)	84 (14.0)	1082 (13.9)
Diabetes	1241 (3.4)	435 (4.6)	265 (2.1)	47 (3.4)	157 (6.7)	58 (2.8)	31 (5.2)	248 (3.2)
Dyslipidemia	1300 (3.6)	357 (3.7)	356 (2.8)	82 (5.9)	105 (4.5)	89 (4.3)	21 (3.5)	290 (3.7)
Angina and myocardial infarction	447 (1.2)	188 (2.0)	87 (0.7)	20 (1.4)	46 (2.0)	22 (1.1)	11 (1.8)	73 (0.9)
Transient ischemic attack or cerebral infarction	214 (0.6)	93 (1.0)	44 (0.3)	12 (0.9)	23 (1.0)	8 (0.4)	1 (0.2)	33 (0.4)
Chronic kidney disease	35 (0.1)	12 (0.1)	7 (0.1)	2 (0.1)	3 (0.1)	4 (0.2)	0	7 (0.1)
Tuberculosis or other mycobacteria	13 (<0.1)	8 (0.1)	1 (<0.1)	0	1 (<0.1)	0	1 (0.2)	2 (<0.1)

Abbreviations: BMI, body mass index (calculated as weight in kilograms divided by height in meters squared); eGFR, estimated glomerular filtration rate.

**Table 2.** Characteristics of Female Participants

Characteristic	Total (n = 41 690)	Not a regular drinker (n = 22 871)	Dominant alcoholic beverage					Mixed alcohol (n = 3392)
			Beer (n = 8791)	Sake (n = 485)	Shochu (n = 782)	Wine (n = 5165)	Whiskey (n = 204)	
Age, mean (SD), y	47.4 (12.8)	48.6 (14.0)	45.1 (11.2)	50.1 (12.5)	47.6 (10.9)	47.5 (10.9)	45.6 (12.3)	44.8 (10.4)
BMI, median (IQR)	20.5 (19.0-22.5)	20.6 (19.0-22.8)	20.4 (19.0-22.2)	21.0 (19.2-23.3)	20.9 (19.3-22.8)	20.2 (18.8-22.0)	21.3 (19.4-23.1)	20.5 (19.0-22.4)
Frequency of alcohol intake, median (IQR), time/wk	0 (0-3.0)	0	3.0 (1.0-5.0)	3.0 (1.0-5.0)	4.0 (1.0-6.0)	3.0 (1.0-5.0)	4.0 (2.0-7.0)	4.0 (2.0-6.0)
Amount of alcohol, No. of participants, drinks/d								
Not a regular drinker	22 871 (54.9)	22 871 (100)	0	0	0	0	0	0
0 to <1	14 424 (34.6)	0	7741 (88.1)	317 (65.4)	452 (57.8)	4246 (82.2)	151 (74.0)	1517 (44.7)
1 to <2	3109 (7.5)	0	810 (9.2)	109 (22.5)	158 (20.2)	730 (14.1)	34 (16.7)	1268 (37.4)
2 to <3	909 (2.2)	0	197 (2.2)	47 (9.7)	97 (12.4)	144 (2.8)	13 (6.4)	411 (12.1)
≥3	377 (0.9)	0	43 (0.5%)	12 (2.5)	75 (9.6)	45 (0.9)	6 (2.9)	196 (5.8)
Amount of total alcohol, mean (SD), drinks/d	0.34 (0.67)	0	0.54 (0.57)	0.94 (0.83)	1.35 (1.34)	0.61 (0.63)	0.86 (1.17)	1.32 (1.08)
Beer	0.16 (0.37)	0	0.54 (0.56)	0.05 (0.16)	0.05 (0.17)	0.01 (0.06)	0.02 (0.15)	0.49 (0.41)
Sake	0.02 (0.16)	0	0 (0.02)	0.88 (0.75)	0 (0.01)	0	0	0.10 (0.35)
Shochu	0.04 (0.29)	0	0 (0.03)	0	1.29 (1.25)	0	0	0.21 (0.54)
Wine	0.11 (0.34)	0	0 (0.02)	0 (0.02)	0.01 (0.06)	0.60 (0.61)	0	0.47 (0.51)
Whiskey	0.01 (0.12)	0	0 (0.02)	0 (0.02)	0 (0.04)	0 (0.02)	0.83 (1.07)	0.05 (0.28)
Serum urate level, mean (SD), mg/dL	4.62 (0.98)	4.59 (0.98)	4.63 (0.97)	4.75 (0.98)	4.79 (1.06)	4.63 (0.96)	4.86 (0.95)	4.72 (0.99)
Kidney function (eGFR), mean (SD), mL/min/1.73 m^2^	97.0 (25.4)	95.6 (26.5)	99.5 (23.7)	97.8 (23.5)	99.2 (24.8)	95.7 (23.5)	104.3 (29.3)	100.6 (23.4)
Medication use indication, No. of participants (%)								
Hypertension	2641 (6.3)	1754 (7.7)	360 (4.1)	54 (11.1)	53 (6.8)	233 (4.5)	11 (5.4)	176 (5.2)
Diabetes	477 (1.1)	377 (1.6)	39 (0.4)	4 (0.8)	11 (1.4)	31 (0.6)	2 (1.0)	13 (0.4)
Dyslipidemia	1296 (3.1)	957 (4.2)	152 (1.7)	17 (3.5)	22 (2.8)	96 (1.9)	7 (3.4)	45 (1.3)
Angina and myocardial infarction	143 (0.3)	113 (0.5)	11 (0.1)	3 (0.6)	0	12 (0.2)	1 (0.5)	3 (0.1)
Transient ischemic attack or cerebral infarction	130 (0.3)	104 (0.5)	9 (0.1)	0	2 (0.3)	13 (0.3)	0	2 (0.1)
Chronic kidney disease	13 (<0.1)	11 (<0.1)	0	1 (0.2)	0	0	0	1 (<0.1)
Tuberculosis or other mycobacteria	43 (0.1)	36 (0.2)	5 (0.1)	0	0	0	0	2 (0.1)

Abbreviations: BMI, body mass index (calculated as weight in kilograms divided by height in meters squared); eGFR, estimated glomerular filtration rate.

### Association of Total Alcohol Consumption and Alcoholic Beverage Consumption With Serum Urate Levels or Hyperuricemia

In a multivariable linear regression model, a 1-unit increase in total alcohol consumption was associated with serum urate levels, with β coefficients of 0.10 mg/dL (95% CI, 0.09-0.11 mg/dL; *P* < .001) for men and 0.14 mg/dL (95% CI, 0.12-0.15 mg/dL; *P* < .001) for women. In a model including all alcoholic beverages, consumption of most beverages showed associations with serum urate levels, whereas sake consumption among women was not significant (β = 0.04 mg/dL [95% CI, −0.01 to 0.09 mg/dL]; *P* = .14). Although ethanol content was standardized, the extent of serum urate variations per consumption of 1 standard drink of each beverage differed. Beer and whiskey were associated with the highest increases in serum urate levels, followed by wine and shochu. Sake was associated with modest increases in serum urate levels among men and women (Table 3). The extent of the association of alcohol consumption with hyperuricemia in multivariable logistic regression was also different among alcoholic beverages (eTable 3 in Supplement 1).

**Table 3.** Association Between Serum Urate Levels and a 1-Unit Increase in Daily Consumption of Total Alcohol and Each Alcoholic Beverage Type

Variable	Men		Women	
	β (95% CI), mg/dL	*P* value	β (95% CI), mg/dL	*P* value
Total alcohol consumption^a^	0.10 (0.09 to 0.11)	<.001	0.14 (0.12 to 0.15)	<.001
Alcoholic beverage type^b^				
Beer	0.12 (0.10 to 0.14)	<.001	0.21 (0.19 to 0.23)	<.001
Sake	0.06 (0.03 to 0.09)	<.001	0.04 (−0.01 to 0.09)	.14
Shochu	0.08 (0.07 to 0.10)	<.001	0.09 (0.06 to 0.12)	<.001
Wine	0.10 (0.07 to 0.13)	<.001	0.10 (0.07 to 0.13)	<.001
Whiskey	0.19 (0.14 to 0.23)	<.001	0.16 (0.10 to 0.23)	<.001

^a^ Adjusted for age, sex, body mass index (calculated as weight in kilograms divided by height in meters squared), estimated glomerular filtration rate, medication use (for hypertension, diabetes, dyslipidemia, angina, myocardial infarction, transient ischemic attack, cerebral infarction, chronic kidney disease, or tuberculosis or other mycobacteria), and results of lifestyle (smoking status, daily physical activity, and exercise level) and dietary questionnaires. Results of lifestyle and dietary questionnaires used as covariates are described in eTables 1 and 2 in Supplement 1.

^b^ Other beverage groups (beer, sake, shochu, wine, and whiskey) were adjusted for consumption of other alcoholic beverages in addition to covariates for total alcohol.

### Association of Serum Urate Levels With Alcohol Consumption for Each Type of Dominant Alcoholic Beverage

We performed multivariable linear regression analyses including an interaction term between daily alcohol consumption and dominant alcoholic beverage type. We observed differences in the association of serum urate levels with ethanol intake among the groups. Whereas alcohol consumption of 1 standard drink in the beer-dominant group was consistently associated with higher serum urate levels for men (0.14 mg/dL [95% CI, 0.11-0.17 mg/dL]; *P* < .001) and women (0.23 mg/dL [95% CI, 0.20-0.26 mg/dL]; *P* < .001), the association in the sake-dominant group was not statistically significant for men (0.05 mg/dL [95% CI, −0.01 to 0.10 mg/dL]; *P* = .10) and women (0.04 mg/dL [95% CI, –0.05 to 0.14 mg/dL]; *P* = .38) (eTable 4 in Supplement 1). Among men, the beer- and whiskey-dominant groups had the steepest slopes in estimated serum urate levels along with alcohol consumption (Figure); the wine-dominant group had an intermediate slope, whereas the other groups had less steep slopes. Among women, the beer-dominant group had the steepest slope in serum urate levels along with alcohol consumption; the wine- and shochu-dominant groups had moderate slopes, whereas the sake- and whiskey-dominant groups had less steep slopes.

**Figure.**
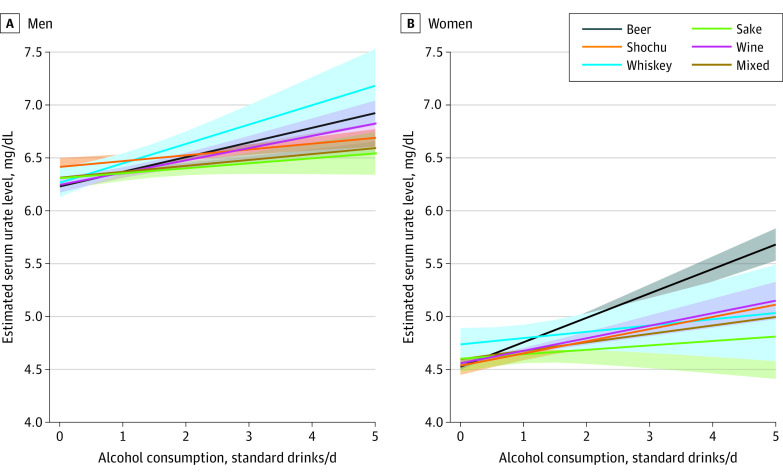
Estimated Serum Urate Levels and Alcohol Consumption for Each Dominant Alcoholic Beverage Group Serum urate levels were estimated using multivariable linear regression including an interaction term between daily alcohol consumption and dominant alcoholic beverage. Lines and shaded areas represent estimated values and 95% CIs, respectively. A standard drink indicates 500 mL of beer, 167 mL (0.93 gou) of sake, 100 mL of shochu, 208 mL of wine, or 62.5 mL of whiskey.

We set a beer-dominant group as a reference for comparison because beer is the most common alcoholic beverage consumed worldwide. Compared with the beer-dominant groups, the model with interaction terms revealed significant differences in the association of serum urate levels with alcohol consumption (per 1 standard drink) among men in the sake-dominant group (−0.09 mg/dL [95% CI, −0.15 to −0.03 mg/dL]; *P* = .004), shochu-dominant group (−0.08 mg/dL [95% CI, −0.12 to −0.05 mg/dL]; *P* < .001), and mixed group (−0.08 mg/dL [95% CI, −0.12 to −0.05 mg/dL]; *P* < .001). For women, significant differences were also observed in all other beverage groups, including sake (−0.19 mg/dL [95% CI, −0.29 to −0.09 mg/dL]; *P* < .001), shochu (−0.12 mg/dL [95% CI, −0.17 to −0.06 mg/dL]; *P* < .001), wine (−0.11 mg/dL [95% CI, −0.16 to −0.06 mg/dL]; *P* < .001), whiskey (−0.17 mg/dL [95% CI, −0.28 to −0.06 mg/dL]; *P* = .002), and mixed (−0.15 mg/dL [95% CI, −0.20 to −0.11 mg/dL]; *P* < .001) (Table 4).

**Table 4.** Comparison of Association of Serum Urate Levels With Dominant Alcoholic Beverage Groups for 1 Standard Drink Increase in Daily Alcohol Consumption

Variable	Men		Women		
	β Coefficient (95% CI), mg/dL^a^	*P* value	β Coefficient (95% CI), mg/dL^a^	*P* value
Alcohol consumption of 1 standard drink/d in beer-dominant group^b^	0.14 (0.11 to 0.17)	<.001	0.23 (0.20 to 0.26)	<.001
Comparison with other beverage groups				
Beer	0 [Reference]	NA	0 [Reference]	NA
Sake	−0.09 (−0.15 to −0.03)	.004	−0.19 (−0.29 to −0.09)	<.001
Shochu	−0.08 (−0.12 to −0.05)	<.001	−0.12 (−0.17 to −0.06)	<.001
Wine	−0.02 (−0.08 to 0.04)	.44	−0.11 (−0.16 to −0.06)	<.001
Whiskey	0.04 (−0.05 to 0.14)	.34	−0.17 (−0.28 to −0.06)	.002
Mixed	−0.08 (−0.12 to −0.05)	<.001	−0.15 (−0.20 to −0.11)	<.001

Abbreviation: NA, not applicable.

^a^ From a multivariable linear regression model including an interaction term between daily alcohol consumption and dominant alcoholic beverage. Adjusted for age, sex, body mass index (calculated as weight in kilograms divided by height in meters squared), estimated glomerular filtration rate, medication use (for hypertension, diabetes, dyslipidemia, angina, myocardial infarction, transient ischemic attack, cerebral infarction, chronic kidney disease, or tuberculosis or other mycobacteria), and results of lifestyle (smoking status, daily physical activity, and exercise level) and dietary questionnaires. Results of lifestyle and dietary questionnaires used as covariates are described in eTables 1 and 2 in Supplement 1.

^b^ Standard drink (500 mL of beer, 167 mL [0.93 gou] of sake, 100 mL of shochu, 208 mL of wine, or 62.5 mL of whiskey).

### Modeling of Association of Serum Urate Levels With Alcohol Consumption Using a Restricted Cubic Spline

To examine the degree of departure from the linear association, we developed more flexible models using a restricted cubic spline with 4 percentile knots (5th, 35th, 65th, and 95th percentiles)^27^ for each dominant alcoholic beverage group. The associations were expressed as almost linear. Among men and women, the beer-dominant groups showed an increase in serum urate levels with alcohol consumption. As for the whiskey-dominant group, serum urate levels were associated with alcohol consumption among men, but there was no such trend among women (with wide 95% CIs). In the sake-dominant group, the slope was less steep, particularly in women (eFigure 2 in Supplement 1). We also used a restricted cubic spline with fixed knots (1, 2, 3, or 4 drinks per day) for each dominant alcoholic beverage group in sensitivity analyses. Trends in slopes were similar to those with percentile knots for men and women (eFigure 3 in Supplement 1).

## Discussion

In this cross-sectional study, we observed differences in the association of serum urate levels with consumption of various alcoholic beverages. Beer and whiskey consumption among men and beer consumption among women were consistently associated with larger increases in serum urate levels than other alcoholic beverages. In contrast, sake consistently showed a modest elevation of serum urate levels with increased intake. In this study, the association of serum urate levels with beer consumption was approximately 2 to 5 times that with sake.

Ethanol in alcoholic beverages is known as a major component that elevates serum urate levels by increasing uric acid production and decreasing urinary excretion of uric acid.^14,16^ In previous studies, the ethanol content in 1 unit of alcoholic beverage differed.^18,28,29^ In this study, we converted the unit of each alcoholic beverage to a standard drink containing the same ethanol content, allowing for comparison with a focus on alcoholic beverage type. Beer intake was associated with higher serum urate levels in this study, as also shown in previous studies.^11,18,30^ As for sake, only a few studies from Japan^19,20^ concluded that sake consumption was also associated with hyperuricemia. The outcome used in those studies was hyperuricemia (serum urate level ≥7 mg/dL), which was different from the outcome of serum urate levels as a continuous variable in this study. In addition, confounding by dietary factors was not adjusted in these studies. Although the association of serum urate levels with wine consumption is under debate,^11,18,30^ this study supports the idea that wine consumption is associated with a substantial but milder increase in serum urate levels. We observed that whiskey consumption was consistently associated with high serum urate levels in men; the same trend was observed in the analyses without dominant alcoholic beverage groups in women. These results were compatible with those from previous studies.^11,18,30^ However, the results from analyses including an interaction term in women were inconsistent, which might be explained by the decreased number of female whiskey-dominant drinkers when dominant beverage groups were created.

Considering that ethanol content was standardized in this study, the difference in serum urate change may be explained by variation in other factors among beverages. In addition to ethanol, other components such as purines, which are metabolized to uric acid, are known to influence serum urate levels.^17^ Beer contains the highest amount of purines, while other beverages include small but differing amounts.^31^ Moreover, total energy intake may be associated with serum urate levels via obesity, and beer usually has higher energy than other beverages.^26,32,33^ Other components—such as ingredients with antioxidant properties, like polyphenols—may have a potential role in ameliorating serum urate levels, since serum urate can increase in response to oxidative stress.^18^ Antioxidants such as polyphenols are included predominantly in wine^34^; sake also includes other antioxidants such as ferulic acid, which may be associated with sake intake and serum urate levels. Furthermore, the gut microbiota can potentially mediate between alcohol consumption and changes in serum urate levels. A previous study suggests that both acute and chronic alcohol consumption can modify microbiome composition.^35^ Beer was associated with changes in gut microbiome variety in a Japanese cohort.^36^ An association of gut microbiome variety with serum urate levels has also been observed.^37^ While our study showed an association between serum urate levels and whiskey consumption among men as reported in previous studies,^11,18^ whiskey contains only a small amount of purines. One study even suggested a urate-lowering property of whiskey in the short term.^38^ Further studies investigating physiologic mechanisms are needed. Because individuals who purchase different alcoholic beverages tend to buy different foods,^39,40^ we should also consider residual confounding, particularly for dietary habits, which might not be fully adjusted by the dietary questionnaire.

Previous studies revealed that genetic variance plays an important role in the development of hyperuricemia^41^ and its influence can be larger than that of dietary factors.^11^ Dietary factors are still important because they are modifiable.^42^ Dietary modification may be considered as a potential intervention, although the effects seem modest, in addition to pharmacotherapy with urate-lowering medications.^32^ Considering that genetic variance plays an important role in hyperuricemia, there may be a specific population for which alcohol consumption contributes to greater increases in serum urate levels. Approaches to the high-risk population with consideration of gene-diet interactions should be applied in the future.

### Strengths and Limitations

This study has several strengths. First, to our knowledge, this is the largest study to analyze the association of serum urate levels with alcohol consumption. This study also used flexible modeling with a spline, suggesting an almost linear association of serum urate levels with alcohol consumption. In addition, we included various types of alcoholic beverages, including sake (Japanese rice wine), which is becoming more popular worldwide. Finally, we directly compared serum urate levels in association with alcoholic beverages by establishing dominant alcoholic beverage groups and using interaction analyses.

This study also has several limitations. In this single-center study, our target population was participants who had received medical checkups, and most lived in urban areas in Japan. These aspects of the study can limit the generalizability of our findings. The questionnaires used in these medical checkups were not standardized instruments such as food frequency questionnaires.^28^ The self-report questionnaire might cause misclassification, which could differ by alcoholic beverage type. This is a cross-sectional study, and we did not directly examine how changing the amount of alcohol intake or switching the dominant drink may impact future serum urate levels. Additionally, there may be unadjusted confounders such as potential variations in dietary habits.

## Conclusions

In this cross-sectional study, there were differences in the association of serum urate levels with alcohol intake among various types of alcoholic beverages, even when ethanol intake was standardized. Beer was associated with increased serum urate levels among both men and women. In contrast, sake consistently had a modest influence. In addition to the amount of total alcohol consumption, the type of alcoholic beverage was associated with serum urate levels.

## Notes

Supplement 1. **eTable 1.** Lifestyle Questionnaire Results for Men **eTable 2.** Lifestyle Questionnaire Results for Women **eTable 3.** Association of Hyperuricemia With a 1-Unit Increase by Daily Total Alcohol Consumption and Alcoholic Beverage Type **eTable 4.** Extent of Association of Serum Urate Levels With Alcohol Consumption of 1 Standard Drink in Each Dominant Beverage Group in Multivariable Linear Regression Analyses **eFigure 1.** Flowchart of Participant Inclusion and Exclusion **eFigure 2.** Estimated Serum Urate Levels and Alcohol Consumption Among Men and Women in Each Dominant Alcoholic Beverage Group Using a Restricted Cubic Spline With Percentile (5th, 35th, 65th, and 95th) Knots **eFigure 3.** Estimated Serum Urate Levels and Alcohol Consumption Among Men and Women for Each Dominant Alcoholic Beverage Type Using a Restricted Cubic Spline With Fixed (1, 2, 3, and 4 Drinks per Day) Knots

Supplement 2.
Data Sharing Statement
