# Supplementary material for: Differences in the Association Between Alcoholic Beverage Type and Serum Urate Levels Using Standardized Ethanol Content
Source: JAMA Netw Open. 2023 Mar 17;6(3):e233398. doi: 10.1001/jamanetworkopen.2023.3398 (PMC10024203; doi:10.1001/jamanetworkopen.2023.3398)
Supplement: Supplement 1. — eTable 1. Lifestyle Questionnaire Results for Men eTable 2. Lifestyle Questionnaire Results for Women eTable 3. Association of Hyperuricemia With a 1-Unit Increase by Daily Total Alcohol Consumption and Alcoholic Beverage Type eTable 4. Extent of Association of Serum Urate Levels With Alcohol Consumption of 1 Standard Drink in Each Dominant Beverage Group in Multivariable Linear Regression Analyses eFigure 1. Flowchart of Participant Inclusion and Exclusion eFigure 2. Estimated Serum Urate Levels and Alcohol Consumption Among Men and Women in Each Dominant Alcoholic Beverage Group Using a Restricted Cubic Spline With Percentile (5th, 35th, 65th, and 95th) Knots eFigure 3. Estimated Serum Urate Levels and Alcohol Consumption Among Men and Women for Each Dominant Alcoholic Beverage Type Using a Restricted Cubic Spline With Fixed (1, 2, 3, and 4 Drinks per Day) Knots [file jamanetwopen-e233398-s001.pdf]

Supplementary Online Content

Fukui S, Okada M, Rahman M, et al. Differences in the association between alcoholic beverage type and serum urate levels using standardized ethanol content. *JAMA Netw Open*. 2023;6(3):e233398. doi:10.1001/jamanetworkopen.2023.3398

eTable 1. Lifestyle Questionnaire Results for Men
eTable 2. Lifestyle Questionnaire Results for Women
eTable 3. Association of Hyperuricemia With a 1-Unit Increase by Daily Total Alcohol Consumption and Alcoholic Beverage Type
eTable 4. Extent of Association of Serum Urate Levels With Alcohol Consumption of 1 Standard Drink in Each Dominant Beverage Group in Multivariable Linear Regression Analyses
eFigure 1. Flowchart of Participant Inclusion and Exclusion
eFigure 2. Estimated Serum Urate Levels and Alcohol Consumption Among Men and Women in Each Dominant Alcoholic Beverage Group Using a Restricted Cubic Spline With Percentile (5th, 35th, 65th, and 95th) Knots
eFigure 3. Estimated Serum Urate Levels and Alcohol Consumption Among Men and Women for Each Dominant Alcoholic Beverage Type Using a Restricted Cubic Spline With Fixed (1, 2, 3, and 4 Drinks per Day) Knots

This supplementary material has been provided by the authors to give readers additional information about their work.

© 2023 Fukui S et al. *JAMA Network Open*.

eTable 1. Lifestyle Questionnaire Results for Men

Lifestyle		No. (%) Total N=36,463	No regular drinker N=9,527	Beer dominant N=12,711	Sake dominant N=1,384	Shochu dominant N=2,343	Wine dominant N=2,088	Whiskey dominant N=600	Mixed N=7,810
Smoke	None	16,484 (45.2)	5,194 (54.5)	6,071 (47.8)	481 (34.8)	559 (23.9)	1,121 (53.7)	174 (29.0)	2,884 (36.9)
	Previous	12,818 (35.2)	2,730 (28.7)	4,126 (32.5)	647 (46.7)	1,011 (43.1)	752 (36.0)	242 (40.3)	3,310 (42.4)
	Current	7,161 (19.6)	1,603 (16.8)	2,514 (19.8)	256 (18.5)	773 (33.0)	215 (10.3)	184 (30.7)	1,616 (20.7)
Daily Physical Activity	Very low	4,819 (13.2)	1,533 (16.1)	1,743 (13.7)	133 (9.6)	251 (10.7)	203 (9.7)	81 (13.5)	875 (11.2)
	Low	13,561 (37.2)	3,720 (39.0)	4,858 (38.2)	442 (31.9)	833 (35.6)	746 (35.7)	207 (34.5)	2,755 (35.3)
	Moderate	15,138 (41.5)	3,589 (37.7)	5,210 (41.0)	661 (47.8)	1,064 (45.4)	922 (44.2)	252 (42.0)	3,440 (44.0)
	High	2,945 (8.1)	685 (7.2)	900 (7.1)	148 (10.7)	195 (8.3)	217 (10.4)	60 (10.0)	740 (9.5)
Exercise	Almost none	11,364 (31.2)	3,434 (36.0)	4,258 (33.5)	324 (23.4)	664 (28.3)	450 (21.6)	186 (31.0)	2,048 (26.2)
	1-2 days / week	14,880 (40.8)	3,482 (36.5)	5,326 (41.9)	540 (39.0)	967 (41.3)	849 (40.7)	233 (38.8)	3,483 (44.6)
	3-5 days / week	6,291 (17.3)	1,539 (16.2)	2,013 (15.8)	307 (22.2)	414 (17.7)	500 (23.9)	110 (18.3)	1,408 (18.0)
	Almost everyday	3,928 (10.8%)	1,072 (11.3%)	1,114 (8.8%)	213 (15.4%)	298 (12.7%)	289 (13.8%)	71 (11.8%)	871 (11.2%)
Diet									
Carbohydrate	Less than 1 meal/day	707 (1.9)	130 (1.4)	183 (1.4)	22 (1.6)	84 (3.6)	87 (4.2)	42 (7.0)	159 (2.0)
	For 1 meal / day	5,047 (13.8)	962 (10.1)	1,569 (12.3)	200 (14.5)	448 (19.1)	421 (20.2)	118 (19.7)	1,329 (17.0)
	For 2 meal / day	9,627 (26.4)	2,048 (21.5)	3,348 (26.3)	392 (28.3)	700 (29.9)	588 (28.2)	141 (23.5)	2,410 (30.9)
	For every meal	21,079 (57.8)	6,386 (67.0)	7,610 (59.9)	770 (55.6)	1,111 (47.4)	991 (47.5)	299 (49.8)	3,912 (50.1)
Meat and Eggs	Almost none	356 (1.0)	172 (1.8)	66 (0.5)	13 (0.9)	21 (0.9)	24 (1.1)	9 (1.5)	51 (0.7)
	1-2 days / week	8,030 (22.0)	2,174 (22.8)	2,604 (20.5)	367 (26.5)	608 (25.9)	457 (21.9)	123 (20.5)	1,697 (21.7)
	3-4 days / week	20,455 (56.1)	5,085 (53.4)	7,387 (58.1)	756 (54.6)	1,275 (54.4)	1,168 (56.0)	304 (50.7)	4,480 (57.4)
	>= 5 days / week	7,619 (20.9)	2,095 (22.0)	2,653 (20.9)	248 (17.9)	439 (18.7)	438 (21.0)	164 (27.3)	1,582 (20.3)
Seafood	Almost none	2,122 (5.8)	909 (9.5)	711 (5.6)	31 (2.2)	95 (4.1)	94 (4.5)	45 (7.5)	237 (3.0)
	1-2 days / week	17,197 (47.2)	4,858 (51.0)	6,525 (51.3)	422 (30.5)	973 (41.5)	884 (42.4)	283 (47.2)	3,252 (41.6)
	3-4 days / week	14,559 (39.9)	3,232 (33.9)	4,833 (38.0)	721 (52.1)	1,025 (43.7)	905 (43.4)	227 (37.8)	3,616 (46.3)
	>= 5 days / week	2,582 (7.1)	527 (5.5)	641 (5.0)	210 (15.2)	250 (10.7)	204 (9.8)	45 (7.5)	705 (9.0)
Vegetables	Less than 1 meal/day	1,692 (4.6)	584 (6.1)	601 (4.7)	39 (2.8)	120 (5.1)	40 (1.9)	37 (6.2)	271 (3.5)
	1 meal / day	15,518 (42.6)	4,098 (43.0)	5,761 (45.3)	486 (35.1)	976 (41.7)	704 (33.7)	262 (43.7)	3,231 (41.4)
	2 meal / day	10,017 (27.5)	2,406 (25.3)	3,507 (27.6)	422 (30.5)	613 (26.2)	641 (30.7)	154 (25.7)	2,274 (29.1)
	Every meal	9,233 (25.3)	2,438 (25.6)	2,841 (22.4)	437 (31.6)	634 (27.1)	702 (33.6)	147 (24.5)	2,034 (26.0)
Fatty Diet	Almost none	4,776 (13.1)	1,433 (15.0)	1,283 (10.1)	279 (20.2)	349 (14.9)	464 (22.2)	66 (11.0)	902 (11.5)
	Sometimes	24,134 (66.2)	6,006 (63.1)	8,507 (66.9)	933 (67.5)	1,576 (67.3)	1,311 (62.8)	400 (66.7)	5,401 (69.2)
	Often	7,548 (20.7)	2,086 (21.9)	2,920 (23.0)	171 (12.4)	418 (17.8)	312 (14.9)	134 (22.3)	1,507 (19.3)
Sweets	Almost none	8,123 (22.3)	1,214 (12.7)	2,468 (19.4)	416 (30.1)	853 (36.4)	657 (31.5)	202 (33.7)	2,313 (29.6)
	1-2 days / week	13,644 (37.4)	3,246 (34.1)	4,863 (38.3)	525 (37.9)	923 (39.4)	754 (36.1)	203 (33.8)	3,130 (40.1)

© 2023 Fukui S et al. *JAMA Network Open*.

Lifestyle		No. (%) Total N=36,463	No regular drinker N=9,527	Beer dominant N=12,711	Sake dominant N=1,384	Shochu dominant N=2,343	Wine dominant N=2,088	Whiskey dominant N=600	Mixed N=7,810
	3-4 days / week	8,745 (24.0)	2,755 (28.9)	3,284 (25.8)	258 (18.6)	361 (15.4)	427 (20.5)	122 (20.3)	1,538 (19.7)
	>= 5 days / week	5,951 (16.3)	2,312 (24.3)	2,096 (16.5)	185 (13.4)	206 (8.8)	250 (12.0)	73 (12.2)	829 (10.6)
Soy	Almost none	3,609 (9.9)	1,163 (12.2)	1,252 (9.9)	94 (6.8)	200 (8.5)	224 (10.7)	76 (12.7)	600 (7.7)
	1-2 days / week	16,430 (45.1)	4,253 (44.6)	6,125 (48.2)	509 (36.8)	977 (41.7)	866 (41.5)	248 (41.3)	3,452 (44.2)
	3-4 days / week	10,732 (29.4)	2,615 (27.5)	3,635 (28.6)	472 (34.1)	727 (31.0)	616 (29.5)	173 (28.8)	2,494 (31.9)
	>= 5 days / week	5,689 (15.6)	1,495 (15.7)	1,698 (13.4)	309 (22.3)	439 (18.7)	381 (18.3)	103 (17.2)	1,264 (16.2)
Milk and milk product	Almost none	5,544 (15.2)	1,396 (14.7)	1,983 (15.6)	188 (13.6)	466 (19.9)	220 (10.5)	114 (19.0)	1,177 (15.1)
	1-2 cup / week	7,761 (21.3)	1,850 (19.4)	2,886 (22.7)	247 (17.8)	553 (23.6)	387 (18.5)	126 (21.0)	1,712 (21.9)
	3-4 cup / week	7,390 (20.3)	1,762 (18.5)	2,716 (21.4)	267 (19.3)	414 (17.7)	413 (19.8)	119 (19.8)	1,699 (21.8)
	1 cup / day	12,550 (34.4)	3,409 (35.8)	4,186 (32.9)	572 (41.3)	731 (31.2)	804 (38.5)	201 (33.5)	2,647 (33.9)
	1.5 or more / day	3,215 (8.8)	1,109 (11.6)	939 (7.4)	110 (7.9)	179 (7.6)	263 (12.6)	40 (6.7)	575 (7.4)
Fruits	Almost none	8,901 (24.7)	2,044 (21.9)	3,339 (26.5)	274 (20.0)	737 (31.6)	328 (16.1)	215 (36.1)	1,964 (25.3)
	1-2 dishes / week	8,908 (24.7)	2,257 (24.1)	3,278 (26.0)	285 (20.8)	591 (25.4)	390 (19.1)	134 (22.5)	1,973 (25.5)
	3-4 dishes / week	5,510 (15.3)	1,398 (15.0)	1,980 (15.7)	202 (14.7)	311 (13.4)	295 (14.5)	74 (12.4)	1,250 (16.1)
	1 dish / day	10,201 (28.3)	2,800 (30.0)	3,285 (26.0)	470 (34.3)	568 (24.4)	790 (38.8)	142 (23.9)	2,146 (27.7)
	2 dishes or more /day	2,524 (7.0)	849 (9.1)	733 (5.8)	139 (10.1)	122 (5.2)	235 (11.5)	30 (5.0)	416 (5.4)

© 2023 Fukui S et al. *JAMA Network Open*.

eTable 2. Lifestyle Questionnaire Results for Women

		No. (%)							
		Total	No regular drinker	Beer dominant	Sake dominant	Shochu dominant	Wine dominant	Whiskey dominant	Mixed
Life style		N=41,690	N=22,871	N=8,791	N=485	N=782	N=5,165	N=204	N=3,392
Smoke	None	4,358 (10.5)	2,388 (10.4)	956 (10.9)	60 (12.4)	91 (11.6)	468 (9.1)	21 (10.3)	374 (11.0)
	Previous	12,419 (29.8)	6,717 (29.4)	2,652 (30.2)	171 (35.3)	242 (30.9)	1,487 (28.8)	72 (35.3)	1,078 (31.8)
	Current	21,564 (51.7)	11,926 (52.1)	4,484 (51.0)	224 (46.2)	378 (48.3)	2,765 (53.5)	92 (45.1)	1,695 (50.0)
Daily Physical Activity	Very low	3,349 (8.0)	1,840 (8.0)	699 (8.0)	30 (6.2)	71 (9.1)	445 (8.6)	19 (9.3)	245 (7.2)
	Low	16,043 (38.5)	8,934 (39.1)	3,604 (41.0)	185 (38.1)	285 (36.4)	1,672 (32.4)	78 (38.2)	1,285 (37.9)
	Moderate	14,393 (34.5)	7,413 (32.4)	3,149 (35.8)	166 (34.2)	270 (34.5)	2,037 (39.4)	68 (33.3)	1,290 (38.0)
	High	7,550 (18.1)	4,303 (18.8)	1,395 (15.9)	89 (18.4)	160 (20.5)	997 (19.3)	38 (18.6)	568 (16.7)
Exercise	Almost none	3,704 (8.9)	2,221 (9.7)	643 (7.3)	45 (9.3)	67 (8.6)	459 (8.9)	20 (9.8)	249 (7.3)
	1-2 days / week	2,751 (6.6)	1,818 (7.9)	380 (4.3)	56 (11.5)	56 (7.2)	246 (4.8)	12 (5.9)	183 (5.4)
	3-5 days / week	684 (1.6)	523 (2.3)	61 (0.7)	7 (1.4)	19 (2.4)	47 (0.9)	3 (1.5)	24 (0.7)
	Almost everyday	1,475 (3.5)	1,084 (4.7)	174 (2.0)	17 (3.5)	23 (2.9)	117 (2.3)	7 (3.4)	53 (1.6)
Diet									
Carbohydrate	Less than 1 meal/day	618 (1.5)	272 (1.2)	105 (1.2)	12 (2.5)	19 (2.4)	118 (2.3)	17 (8.3)	75 (2.2)
	For 1 meal / day	5,223 (12.5)	2,051 (9.0)	1,243 (14.1)	81 (16.7)	164 (21.0)	937 (18.1)	43 (21.1)	704 (20.8)
	For 2 meal / day	11,761 (28.2)	5,526 (24.2)	2,848 (32.4)	172 (35.5)	254 (32.5)	1,649 (31.9)	76 (37.3)	1,236 (36.4)
	For every meal	24,085 (57.8)	15,020 (65.7)	4,594 (52.3)	220 (45.4)	345 (44.1)	2,461 (47.6)	68 (33.3)	1,377 (40.6)
Meat and eggs	Almost none	599 (1.4)	418 (1.8)	71 (0.8)	13 (2.7)	11 (1.4)	59 (1.1)	3 (1.5)	24 (0.7)
	1-2 days / week	8,009 (19.2)	4,622 (20.2)	1,576 (17.9)	105 (21.6)	141 (18.0)	940 (18.2)	36 (17.6)	589 (17.4)
	3-4 days / week	23,679 (56.8)	12,789 (55.9)	5,127 (58.3)	269 (55.5)	469 (60.0)	2,944 (57.0)	91 (44.6)	1,990 (58.7)
	>= 5 days / week	9,400 (22.5)	5,040 (22.0)	2,016 (22.9)	98 (20.2)	161 (20.6)	1,222 (23.7)	74 (36.3)	789 (23.3)
Seafood	Almost none	2,699 (6.5)	1,704 (7.5)	523 (5.9)	12 (2.5)	31 (4.0)	244 (4.7)	15 (7.4)	170 (5.0)
	1-2 days / week	19,130 (45.9)	10,363 (45.3)	4,309 (49.0)	189 (39.0)	352 (45.0)	2,252 (43.6)	103 (50.5)	1,562 (46.0)
	3-4 days / week	17,066 (40.9)	9,229 (40.4)	3,488 (39.7)	226 (46.6)	337 (43.1)	2,300 (44.5)	69 (33.8)	1,417 (41.8)
	>= 5 days / week	2,792 (6.7)	1,573 (6.9)	470 (5.3)	58 (12.0)	62 (7.9)	369 (7.1)	17 (8.3)	243 (7.2)
Vegetables	Less than 1 meal/day	932 (2.2)	539 (2.4)	208 (2.4)	9 (1.9)	20 (2.6)	79 (1.5)	5 (2.5)	72 (2.1)
	For 1 meal / day	11,705 (28.1)	6,141 (26.9)	2,768 (31.5)	154 (31.8)	266 (34.0)	1,241 (24.0)	65 (31.9)	1,070 (31.5)
	For 2 meal / day	13,657 (32.8)	7,285 (31.9)	2,923 (33.3)	153 (31.5)	261 (33.4)	1,780 (34.5)	63 (30.9)	1,192 (35.1)
	For every meal	15,394 (36.9)	8,905 (38.9)	2,891 (32.9)	169 (34.8)	235 (30.1)	2,065 (40.0)	71 (34.8)	1,058 (31.2)
Fatty Diet	Almost none	9,454 (22.7)	5,624 (24.6)	1,533 (17.4)	123 (25.4)	172 (22.0)	1,343 (26.0)	45 (22.1)	614 (18.1)
	Sometimes	28,123 (67.5)	15,130 (66.2)	6,272 (71.4)	315 (64.9)	515 (65.9)	3,384 (65.5)	127 (62.3)	2,380 (70.2)
	Often	4,111 (9.9)	2,116 (9.3)	985 (11.2)	47 (9.7)	95 (12.1)	438 (8.5)	32 (15.7)	398 (11.7)
Sweets	Almost none	4,703 (11.3)	1,929 (8.4)	1,133 (12.9)	83 (17.1)	149 (19.1)	792 (15.3)	53 (26.0)	564 (16.6)
		No. (%)							

© 2023 Fukui S et al. *JAMA Network Open*.

Life style		Total N=41,690	No regular drinker N=22,871	Beer dominant N=8,791	Sake dominant N=485	Shochu dominant N=782	Wine dominant N=5,165	Whiskey dominant N=204	Mixed N=3,392
	1-2 days / week	11,707 (28.1)	5,969 (26.1)	2,685 (30.5)	153 (31.5)	240 (30.7)	1,505 (29.1)	54 (26.5)	1,101 (32.5)
	3-4 days / week	12,182 (29.2)	6,890 (30.1)	2,532 (28.8)	124 (25.6)	218 (27.9)	1,409 (27.3)	46 (22.5)	963 (28.4)
	>= 5 days / week	13,098 (31.4)	8,083 (35.3)	2,441 (27.8)	125 (25.8)	175 (22.4)	1,459 (28.2)	51 (25.0)	764 (22.5)
Soy	Almost none	2,439 (5.9)	1,322 (5.8)	519 (5.9)	29 (6.0)	47 (6.0)	310 (6.0)	17 (8.3)	195 (5.7)
	1-2 days / week	14,195 (34.1)	7,467 (32.7)	3,249 (37.0)	181 (37.3)	279 (35.7)	1,690 (32.7)	74 (36.3)	1,255 (37.0)
	3-4 days / week	15,510 (37.2)	8,479 (37.1)	3,310 (37.7)	176 (36.3)	278 (35.5)	1,915 (37.1)	66 (32.4)	1,286 (37.9)
	>= 5 days / week	9,543 (22.9)	5,601 (24.5)	1,712 (19.5)	99 (20.4)	178 (22.8)	1,250 (24.2)	47 (23.0)	656 (19.3)
Milk (Product)	Almost none	3,030 (7.3)	1,557 (6.8)	683 (7.8)	45 (9.3)	85 (10.9)	326 (6.3)	36 (17.6)	298 8.8)
	1-2 cup / week	6,315 (15.1)	3,122 (13.7)	1,534 (17.5)	97 (20.0)	147 (18.8)	758 (14.7)	31 (15.2)	626 (18.5)
	3-4 cup / week	9,126 (21.9)	4,635 (20.3)	2,075 (23.6)	109 (22.5)	185 (23.7)	1,189 (23.0)	47 (23.0)	886 (26.1)
	1 cup / day	17,797 (42.7)	10,137 (44.3)	3,611 (41.1)	180 (37.1)	272 (34.8)	2,251 (43.6)	69 (33.8)	1,277 (37.6)
	1.5 or more / day	5,419 (13.0)	3,418 (14.9)	887 (10.1)	54 (11.1)	93 (11.9)	641 (12.4)	21 (10.3)	305 (9.0)
Fruits	Almost none	6,056 (14.8)	2,613 (11.7)	1,652 (19.0)	102 (21.4)	188 (24.4)	688 (13.6)	64 (31.8)	749 (22.4)
	1-2 dishes / week	7,784 (19.1)	3,874 (17.4)	1,936 (22.3)	98 (20.6)	184 (23.9)	886 (17.5)	46 (22.9)	760 (22.7)
	3-4 dishes / week	6,843 (16.7)	3,650 (16.4)	1,552 (17.9)	69 (14.5)	130 (16.9)	821 (16.2)	29 (14.4)	592 (17.7)
	1 dish / day	15,202 (37.2)	8,894 (39.9)	2,872 (33.1)	144 (30.3)	220 (28.6)	2,038 (40.2)	49 (24.4)	985 (29.4)
	2 dishes or more /day	4,973 (12.2)	3,273 (14.7)	675 (7.8)	63 (13.2)	47 (6.1)	637 (12.6)	13 (6.5)	265 (7.9)

© 2023 Fukui S et al. *JAMA Network Open*.

eTable 3. Association of Hyperuricemia With a 1-Unit Increase by Daily Total Alcohol Consumption and Alcoholic Beverage Type

	Male		Female	
Variables	Odds ratio (95% CI)	p value	Odds ratio (95% CI)	p value
Total alcohol [a]	1.18 (1.16, 1.21)	<0.001	1.33 (1.26, 1.39)	<0.001
Beer [b]	1.23 (1.19, 1.28)	<0.001	1.58 (1.45, 1.71)	<0.001
Sake [b]	1.12 (1.06, 1.18)	<0.001	1.03 (0.85, 1.26)	0.75
Shochu [b]	1.15 (1.12, 1.18)	<0.001	1.27 (1.16, 1.39)	<0.001
Wine [b]	1.18 (1.11, 1.25)	<0.001	1.22 (1.10, 1.34)	<0.001
Whiskey [b]	1.37 (1.26, 1.50)	<0.001	1.24 (1.02, 1.52)	0.035

[a] Adjusted for age, sex, BMI, eGFR, medication use (for hypertension, diabetes, dyslipidemia, angina and myocardial infarction, transient ischemic attack or cerebral infarction, chronic kidney disease, and tuberculosis or other mycobacteria), and results of lifestyle questionnaires (smoking status, daily physical activity, exercise level) and dietary questionnaire. Results of the lifestyle and dietary questionnaire used as covariates were all described in Supplemental Tables 1 and 2.

[b] Adjusted for consumption of other alcoholic beverages in addition to covariates above

© 2023 Fukui S et al. *JAMA Network Open*.

eTable 4. Extent of Association of Serum Urate Levels With Alcohol Consumption of 1 Standard Drink in Each Dominant Beverage Group in Multivariable Linear Regression Analyses

	Male		Female	
Group	Marginal effect (95% CI) [a]	p value	Marginal effect (95% CI) [a]	p value
Beer	0.14 (0.11, 0.17)	< 0.001	0.23 (0.20, 0.26)	< 0.001
Sake	0.05 (-0.01, 0.10)	0.10	0.04 (-0.05, 0.14)	0.38
Shochu	0.05 (0.03, 0.08)	< 0.001	0.11 (0.07, 0.16)	< 0.001
Wine	0.12 (0.06, 0.17)	< 0.001	0.12 (0.08, 0.16)	< 0.001
Whiskey	0.18 (0.10, 0.27)	< 0.001	0.06 (-0.05, 0.16)	0.27
Mixed	0.06 (0.04, 0.07)	< 0.001	0.08 (0.05, 0.11)	< 0.001

[a] These results were from a multivariable linear regression model including an interaction term between daily alcohol consumption and dominant alcohol beverage. Results were adjusted for age, sex, BMI, eGFR, medication use (for hypertension, diabetes, dyslipidemia, angina and myocardial infarction, transient ischemic attack or cerebral infarction, chronic kidney disease, and tuberculosis or other mycobacteria), and results of lifestyle questionnaires (smoking status, daily physical activity, exercise level) and dietary questionnaire. The all results of the lifestyle and dietary questionnaire used as covariates were described in Supplemental Tables 1 and 2.

CI, Confidence interval

© 2023 Fukui S et al. *JAMA Network Open*.

eFigure 1. Flowchart of Participant Inclusion and Exclusion

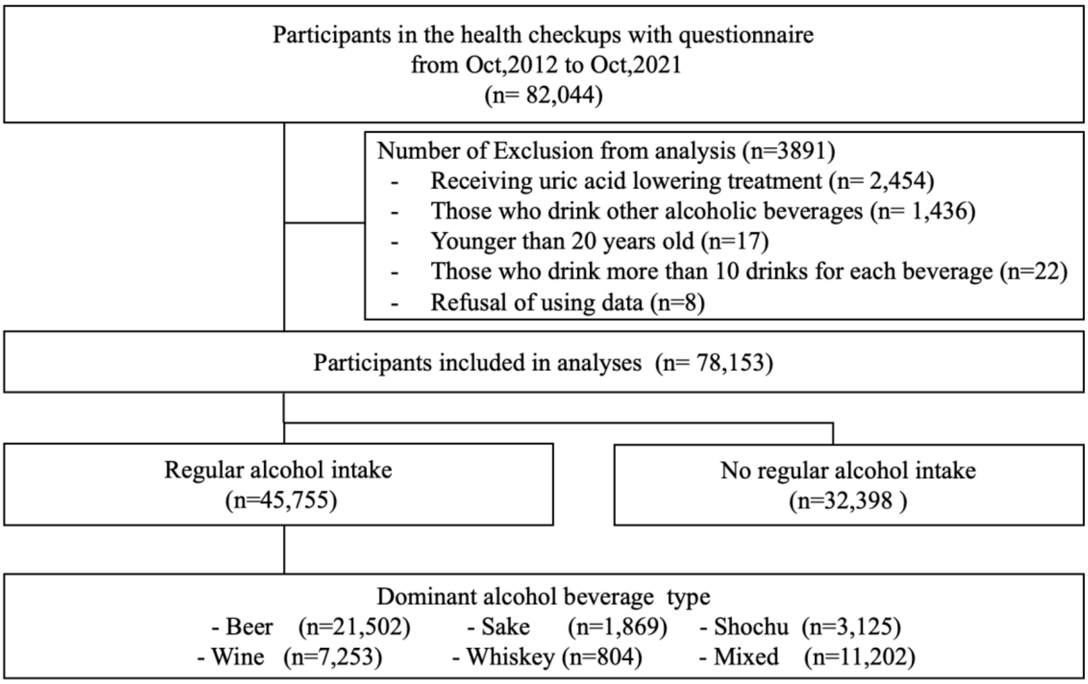

© 2023 Fukui S et al. *JAMA Network Open*.

eFigure 2. Estimated Serum Urate Levels and Alcohol Consumption Among Men and Women in Each Dominant Alcoholic Beverage Group Using a Restricted Cubic Spline With Percentile (5th, 35th, 65th, and 95th) Knots

(A) Men and (B) Women

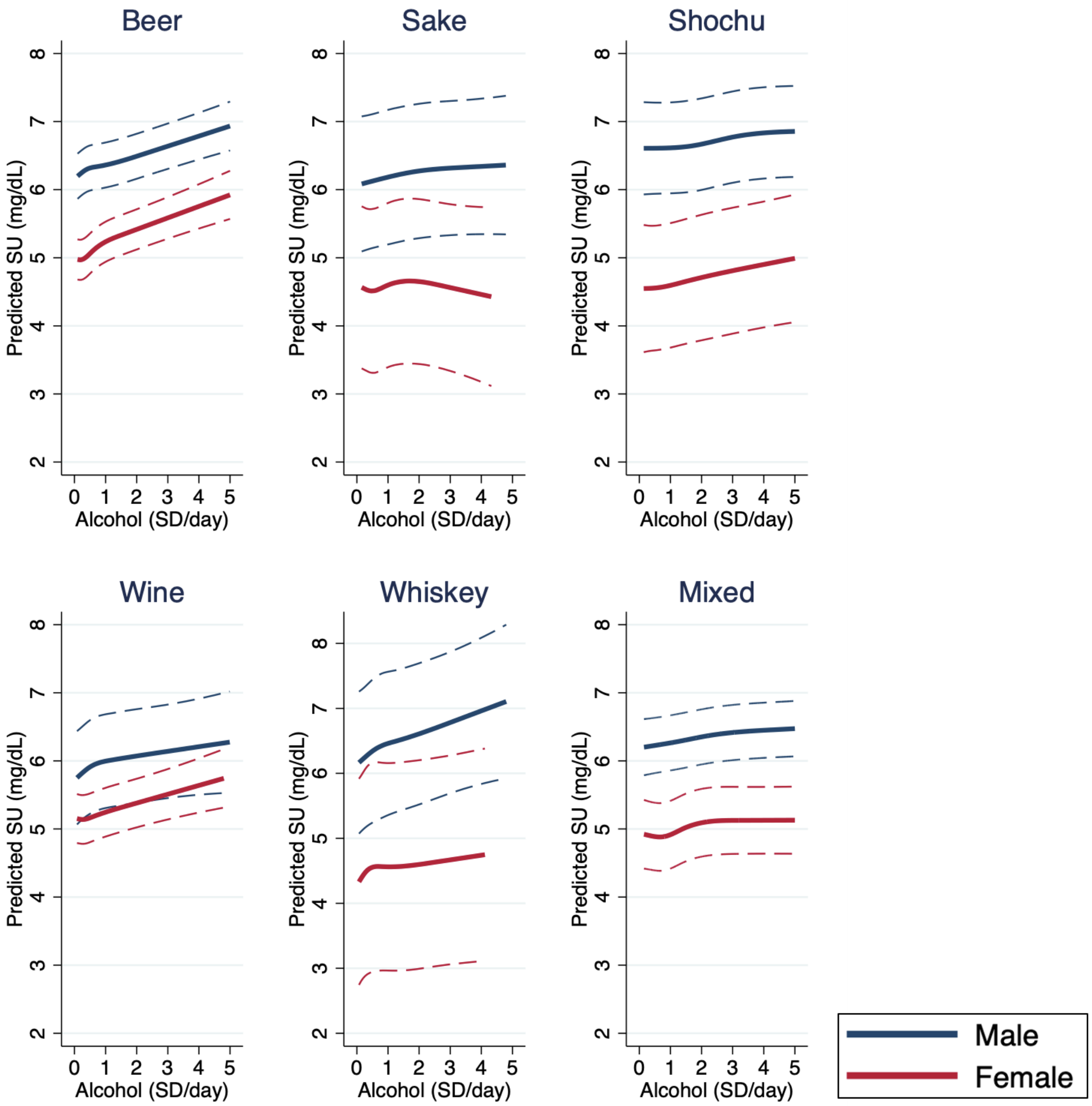

Dashed lines represent the 95% pointwise confidence intervals. SU levels were predicted using multivariable linear regression including an interaction term between daily alcohol consumption and dominant alcohol beverage.

© 2023 Fukui S et al. *JAMA Network Open*.

In each group, 5, 35, 65, and 95 percentiles of alcohol consumption (SD/day) were as follows: Beer (0.1, 0.4, 0.7, 2.2 in males, and 0.1, 0.2, 0.6, 1.5 in females); Sake (0.3, 0.9, 1.9, and 3.4 in males and 0.2, 0.5, 0.9, and 2.7 in females); Shochu (0.3, 1.7, 3.0, and 5.3 in males and 0.1, 0.6, 1.4, and 4.0 in females); Wine (0.1, 0.5, 1.2, and 2.9 in male and 0.1, 0.2, 0.6, and 1.7 in female); Whiskey (0.1, 0.6, 1.2, and 3.1 in males and 0.1, 0.3, 0.8, and 2.4 in females); and mixed (0.4, 1.3, 2.1, and 4.4 in males, 0.2, 0.8, 1.4, and 3.1 in females).

SU, serum urate SD, standard drink (500 ml of beer, 167 ml (0.93gou) of sake, 100 ml of shochu, 208 ml of wine, and 62.5 ml of whiskey.)

© 2023 Fukui S et al. *JAMA Network Open*.

eFigure 3. Estimated Serum Urate Levels and Alcohol Consumption Among Men and Women for Each Dominant Alcoholic Beverage Type Using a Restricted Cubic Spline With Fixed Knots (1, 2, 3, and 4 Drinks per Day)

(A) Men and (B) Women

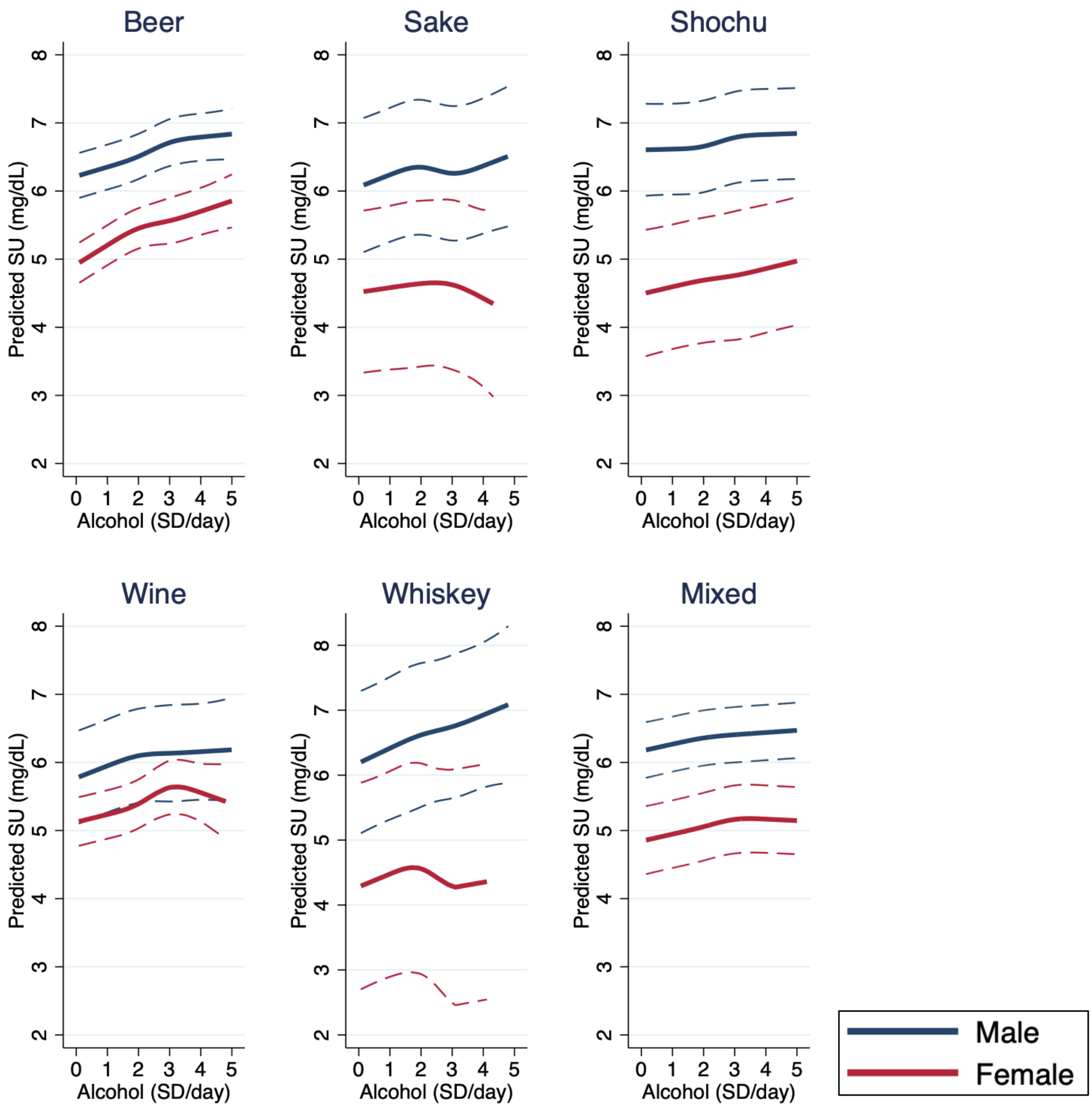

Dashed lines represent the 95% pointwise confidence intervals. SU levels were predicted using multivariable linear regression including an interaction term between daily alcohol consumption and dominant alcohol beverage.

SU, serum urate SD, standard drink (500 ml of beer, 167 ml (0.93gou) of sake, 100 ml of shochu, 208 ml of wine, and 62.5 ml of whiskey.)

© 2023 Fukui S et al. *JAMA Network Open*.
