# Supplementary material for: Differences in the Association Between Alcoholic Beverage Type and Serum Urate Levels Using Standardized Ethanol Content
Source: JAMA Netw Open. 2023 Mar 17;6(3):e233398. doi: 10.1001/jamanetworkopen.2023.3398 (PMC10024203; doi:10.1001/jamanetworkopen.2023.3398)
Supplement: Supplement 2. — Data Sharing Statement [file jamanetwopen-e233398-s002.pdf]

Data Sharing Statement

Fukui. Differences in the Association Between Alcoholic Beverage Type and Serum Urate Levels Using Standardized Ethanol Content. *JAMA Netw Open*. Published March 17, 2023. doi:10.1001/jamanetworkopen.2023.3398

Data
Data available: No

Additional Information
Explanation for why data not available: Data is not publicly available due to the regulation in our institution. Data can be available on reasonable request from the corresponding author (SF) only after approval from the institution.
